# Physiological changes throughout an insect ear due to age and noise - A longitudinal study

**DOI:** 10.1016/j.isci.2022.104746

**Published:** 2022-07-21

**Authors:** Alix Blockley, Daisy Ogle, Charlie Woodrow, Fernando Montealegre-Z, Ben Warren

**Affiliations:** 1 College of Life Sciences, University of Leicester, Leicester LE1 7RH, UK; 2 School of Life Sciences, University of Lincoln, Joseph Banks Laboratories, Lincoln LN6 7DL, UK

**Keywords:** Entomology, Ethology, Cellular neuroscience, Sensory neuroscience

## Abstract

Hearing loss is not unique to humans and is experienced by all animals in the face of wild and eclectic differences in ear morphology. Here, we exploited the high throughput and accessible tympanal ear of the desert locust, *Schistocerca gregaria* to rigorously quantify changes in the auditory system due to noise exposure and age. In this exploratory study, we analyzed tympanal displacements, morphology of the auditory Müller’s organ and measured activity of the auditory nerve, the transduction current, and electrophysiological properties of individual auditory receptors. This work shows that hearing loss manifests as a complex disorder due to differential effects of age and noise on several processes and cell types within the ear. The “middle-aged deafness” pattern of hearing loss found in locusts mirrors that found for humans exposed to noise early in their life suggesting a fundamental interaction of the use of an auditory system (noise) and its aging.

## Highlights

• Locusts routinely exposed to noise follow same pattern of hearing loss as humans
• Parts of the auditory system are affected by noise, age, or both noise and age
• Hearing loss is a multifaceted disorder caused by defects in distinct ear processes

## Graphical abstract

**Figure:**
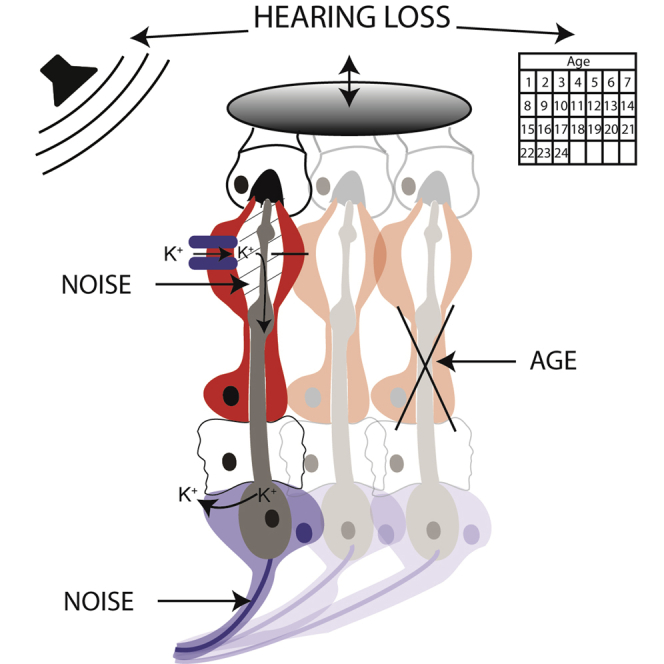

## Introduction

### The multifaceted causes and consequences that lead to hearing loss

Nothing lasts forever. Left unchecked, systems inevitably fail. Two of the biggest causes are age and use. Ears are a fascinating and relevant biological example of this principle. Age-related hearing loss is something that we will inevitably experience and noise-induced hearing loss is responsible for an estimated third of all worldwide hearing loss (Bethesda, 1990). Audiometric measurements from workers in consistently noisy environments—before the enforcement of ear protection—have gifted us a crucial quantitative and longitudinal dataset to understand the interaction of noise and age on hearing loss (Burns and Robinson, 1970; Passchier-Vermeer, 1968, 1977). We know that threshold shifts due to noise can, with a small corrective factor (Corso, 1980), be simply added to threshold shifts due to age (Macrae, 1971; Mills et al., 1998; Corso et al., 1976; Mollica, 1969). From this dataset, Corso (1980) found that noise-exposed individuals differed most from their non-noise-exposed counterparts during the middle of their life but are very similar in older life. This is because age-related hearing loss dominates in later life. This simplistic mathematical interaction of age and noise on hearing thresholds is in stark contrast to the complex and insidious physiological deficits found in ears across animal phyla responsible for hearing loss.

The physiological and anatomical changes that co-occur with hearing loss present at multiple levels of the auditory system. This stretches from the middle ear and tympanum (Etholm and Belal, 1974; Ruah et al., 1991; Rolvien et al., 2018), to the inner ear supporting cells (Thorne and Gavin, 1985; Shi and Nuttall, 2003) and auditory receptors (Keithley and Feldman, 1982; Liberman and Beil, 1978; Bohne and Harding, 2000; Wu et al., 2019; Jeng et al., 2020; Boyd-Gibbins et al., 2021) including their synapses to the auditory nerve (Kujawa and Liberman, 2009, 2015; Wu et al., 2019) and the central nervous system where sound is processed (Fetoni et al., 2013). The most profound and best quantified change in the auditory system is loss of hair cells (Coleman, 1976; Keithley and Feldman, 1982; Bhattacharyya and Dayal, 1985; Dayal and Bhattacharyya, 1986; Li and Hultcranz, 1994; Dixon and Arndt, 1971; Pinheiro et al., 1973; Clark et al., 1974; Moody et al., 1978). Loss of hair cells can lead to loss of the spiral ganglion neurons onto which they synapse (Dupont et al., 1993; McFadden et al., 2004; Bohne and Harding, 2000; Johnson and Hawkins, 1972). The auditory nerve formed by the axons of the spiral ganglion neurons and Schwann cell folds decreases in thickness after noise exposure (Pilati et al., 2012; Tagoe et al., 2014; Wan and Corfas, 2017). The complex correlation of multiple physiological deficits with hearing loss appears to have mistakenly led to the cause being assumed to the most consistent deficit—for instance with age-related hearing loss being due to the most common stria vascularis pathology (Ramadan and Schuknecht, 1989; Wu et al., 2020). In short, we lack the experiments and quantitative data to track the progression of known changes in the auditory system and weigh up their contribution to hearing loss.

Adding to this complexity, it is not known which physiological deficits are caused by noise, age, or a combination of both. There are some breakthrough studies that have proportioned specific deficits into noise or age (Kujawa and Liberman, 2006, 2009; Fernandez et al., 2015). Kujawa and Liberman (2009) found that ribbon synapses—those between hair cells and spiral ganglion neurons—sharply decrease in number after noise but appear to be only mildly decreased with age. And Wu et al. (2020) found that most hearing loss can be explained by hair cell loss not stria vascularis degeneration.

At present, high-powered quantification of multiple physiological deficits in one model organism in response to noise or age over the course of its lifespan are rare (Keder et al., 2020). Further to this, age-related hearing loss and noise-induced hearing loss are thought to be very distinct processes (Yang et al., 2015), which could be independent of each other (Corso et al., 1976). In this exploratory work, we rigorously quantify the physiological changes responsible for hearing loss—found across animal phyla—over the lifespan of the desert locust, *Schistocerca gregaria*. Using a high-powered approach, we measure: *in vivo* displacements of the tympanum, the morphology and anatomy of the auditory Müller’s organ, electrophysiological properties of the auditory receptors, and their transduction currents *ex vivo* and *in vivo* sound-induced auditory nerve responses.

## Results

### Gregarious desert locust survival assay

**Figure 1:**
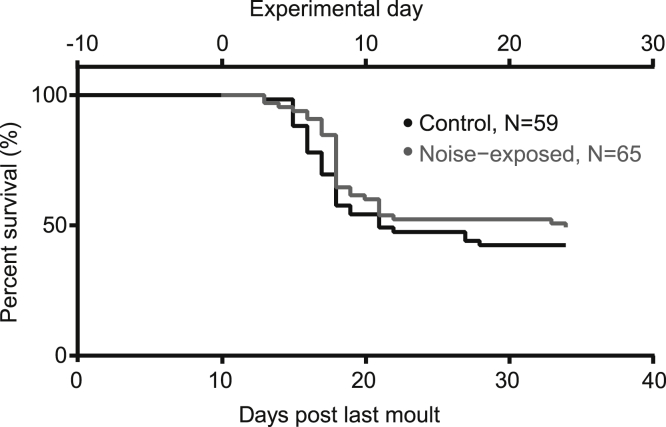
Survival of control and noise-exposed locusts Experimental Day 1 is 10 days post their last moult into adults.

We raised locusts in their fast-aging gregarious state, where they can live up to two months. This contrasts with their solitarious epigenetic state where they live for up to nine months. We counted the deaths of locusts over 34 days (Figure 1). At 10 days post their last moult, the locusts’ wings were clipped and they entered the experimental pipeline. The desert locust follows the same type III (exponential) survival curve (Demetrius, 1978) as other insects (Boll Weevil, Oliveira-Marra et al., 2019; mosquito, Vézilier et al., 2012), where a period of low mortality is followed by a steep exponential decline in survival. Approximately 50% of locusts were dead at 12 days post first noise exposure or mock noise exposure (for control group) and there was no stark difference, in survival, between noise-exposed and control locusts. Although comparing insects’ type III survival curves with mammalian type I curves is a questionable practice, the oldest locusts measured in our study would equate to humans aged 73 years (based on global median life expectancy, UN Data). After this time, the rate of locust mortality decreased drastically so that very few locusts died over the next 12 days until we stopped measuring. There are no quantitative studies that have measured the life expectancy of crowded gregarious desert locusts, only unreferenced reports (Desert Locust Information Service, WMO library).

### Müller’s organ anatomy and function

**Figure 2:**
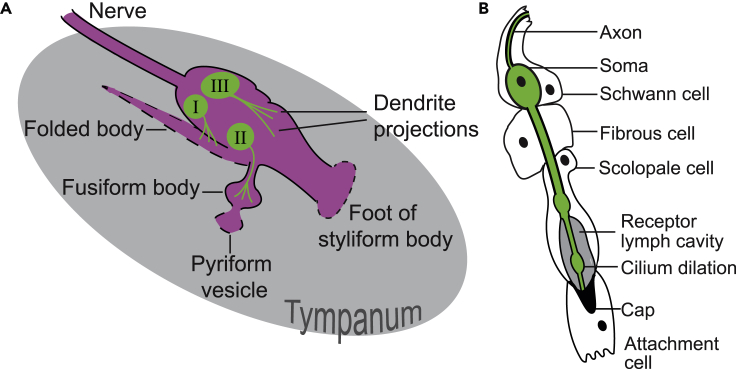
Morphology and cell types of Müller’s organ (A) Müllers organ (purple) is composed of three groups of auditory neurons (green) that attach to distinct parts of the tympanum (gray). (B) Four other cell types enclose various parts of the auditory neurons to make a functional scolopidium.

Müller’s organ is composed of three groups of auditory neurons: ∼46 Group III neurons tuned ∼3 kHz, ∼20 Group I tuned to 0.5–1.5 kHz, and 12–14 Group II tuned between 12 and 25 kHz (Figure 2A) (Jacobs et al., 1999). Hearing thresholds of locusts are as low as 40 dB SPL (Sound Pressure Level) (Michelsen, 1968) and saturate around 110 dB SPL (Warren et al., 2020). We choose to target Group III auditory neurons as these compose the majority of auditory neurons in Müller’s organ and are assessable for single cell electrophysiological recordings. Each group of auditory neurons attaches to distinct parts of the tympanum to exploit the spatial patterns of frequency-specific travelling wave displacements to detect frequencies between 0.2 and 40 kHz. There are another five types of supporting cells that altogether compose Müller’s organ: Schwann cells (that wrap the auditory neuron axons), fibrous cells (that surround the auditory neuron soma), scolopale cells (that engulf the cilium where transduction takes place), and attachment cells that connect the cilium to hypodermal cells that attach to the tympanum (Figure 2B). Desert locusts have no auditory behavior associated with the frequencies to which the majority of their Group III auditory neurons are tuned.

### Doppler laser vibrometry

**Figure 3:**
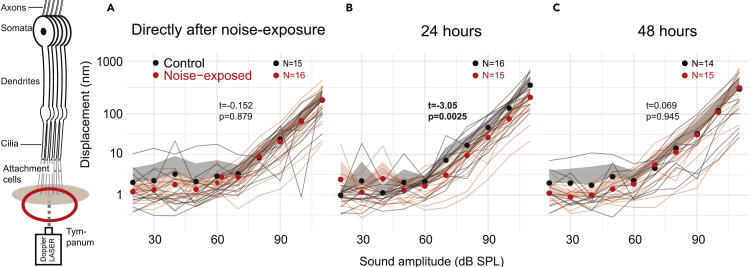
Doppler laser measurements of tone-evoked tympanal displacements (A) Displacements of the tympanum (measured at the foot of the styliform body) in response to 3 kHz pure tones for control locusts and for locusts directly after noise exposure (B) 24 hours after nouse exposure and (C) 48 hours after noise exposure. Individual locusts are plotted as thin gray or thin red lines for control and noise-exposed, respectively. Dots are the average for each treatment at each SPL. The positive standard deviation (negative not shown) is displayed in gray and red shaded areas. The extent of the difference between each group and a statistical test of a difference are displayed as t and p values.

We measured *in vivo* tone-evoked displacements from the external side of the tympanum at the foot of the styliform body, where the majority of group-III auditory neurons attach, using contactless laser Doppler vibrometry. Tone-evoked displacements rose above the noise floor above sound amplitudes of 60 dB SPL (Figures 3A–3C) and higher SPLs remained linear on a log-log plot. Thus, tone-evoked tympanal displacements can be explained by a simple power law. Directly after, and at 48 h after noise exposure, there was no difference in tone-evoked tympanal displacements for control and noise-exposed locusts (Linear Mixed Effects Model (LMEM) with SPL as a random effect; t_(297)_ = −0.152, p = 0.879; t_(209)_ = 0.069, p = 0.945). The tympanal displacements of the noise-exposed locusts 24 h after noise exposure were lower (t_(298)_ = −3.05, p = 0.0025).

### *In vivo* electrophysiology

**Figure 4:**
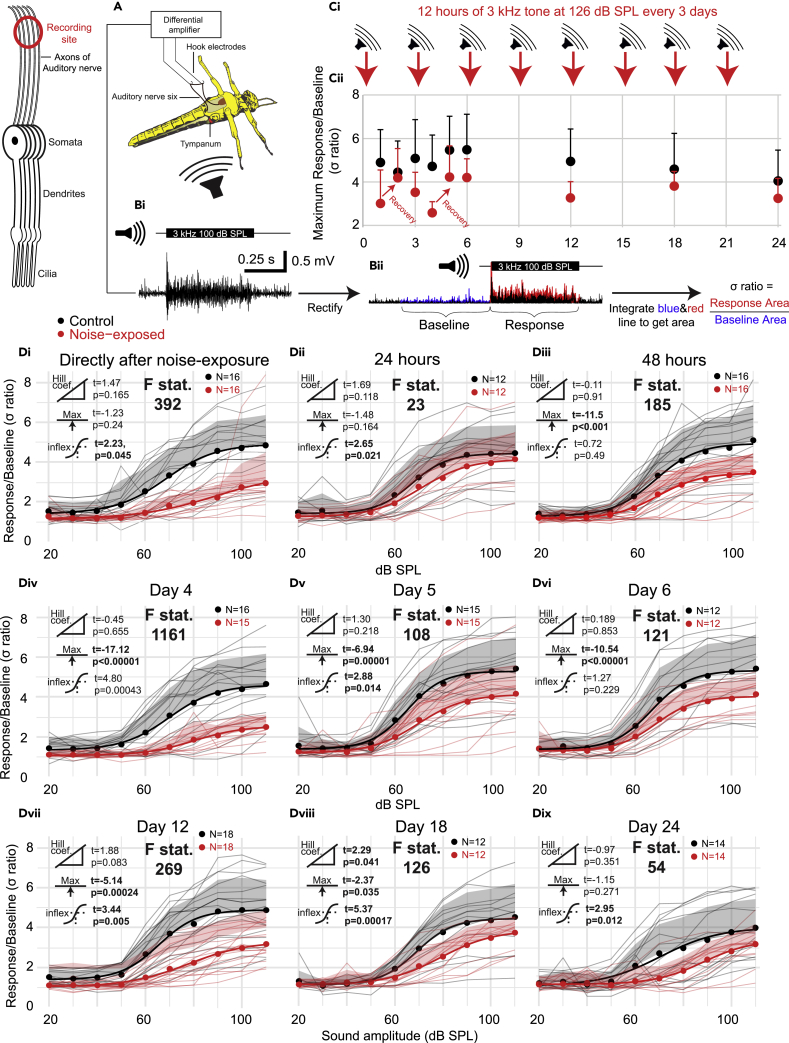
Hook electrode recordings from the auditory nerve quantify noise-induced and age-related hearing loss (A) Schematic of dissection and recording setup. (Bi) Stimulation of 3 kHz pure tone elicits tone-evoked biphasic compound spikes. (Bii) Analytical workflow of tone-evoked responses. Auditory nerve signals were rectified and then the area underneath the tone-evoked signal was divided by the equivalent time and area with no auditory stimulation to get the σ ratio. (Ci) Experimental workflow showing the time of auditory over-exposures and recordings (N.B. days 1 and 4 where directly after noise exposure and days 2 and 5 24 h after noise exposure. Recordings on days 3, 6, 12, 18, and 24 where all 48 h after noise exposure and directly before the next noise exposure). (Cii) Plot of the maximal σ ratio for control and noise-exposed locusts at each recording day with positive standard deviation plotted. (Di) Plot of the σ ratio (nerve response) as a function of SPL of a 3 kHz tone for control and noise-exposed locusts within 12 h after cessation of noise exposure. Responses for individual locusts are plotted as thin gray or thin red lines for control and noise-exposed, respectively. Dots are the average for each treatment at each SPL, shaded area is the positive standard deviation at each SPL, and thick lines are four-parameter Log-Linear fits for each treatment. The extent of the difference between each group and a statistical test of a difference are displayed as t and p values next to three icons donating (from top to bottom) Hill coefficient, maximum asymptote, and the inflection point (p values below 0.05 are in bold). The F statistic compares the fit of the model when the treatment of the group (noise-exposed or control) was omitted. Higher F statistics donate a larger effect of treatment. Dii and Diii plot σ ratio against SPL for noise-exposed and control locusts 24 and 48 h after cessation of noise exposure (or silent speaker for control). Div is directly after a second noise exposure and Dv and Dvi 24 and 48 h after the second noise exposure. Dvii, Dviii, and Dix are 48 h after the 4^th^, 6^th^, and 8^th^ noise exposure, respectively.

We quantified the *in vivo* performance of the auditory neurons to produce sound-evoked spikes in response to repeated noise exposure over an extended 24 day period. We recorded spiking activity directly from the auditory nerve (nerve 6) using hook electrodes in response to 3 kHz tones produced by a speaker (Figures 4A and 4B). A 3 kHz tone matched the frequency of our noise exposure and the frequency to which the majority of auditory neurons (Group III neurons) in Müller’s organ are broadly tuned. These recordings left the bilateral ears, in the first abdominal segment, intact (Figure 4A). We exposed locusts to a 126 dB SPL 3 kHz tone overnight for 12 h every three days and recorded nerve activity at days: 1, 2, 3, 4, 5, 6, 12, 18, and 24 (Figure 4C).

The tone-evoked activity of the auditory nerve was composed of biphasic spikes that summated together at tone onset and for loud SPLs. Therefore, to quantify the tone-evoked activity of the auditory nerve, we rectified the tone-evoked signals, integrated the area underneath, and averaged the three tone presentations at each SPL. We then computed the σ ratio by dividing the average area during the 0.5 s tone by the average area over 0.5 s during 60 s of background activity before tone presentations began (Figure 4Bii). This normalizes for differences in background spiking activity and noise of the recording. To maximize the information from the recordings, we fitted four-part Log-Linear equations to model the relationship between the nerve response and SPL (Figure 4Di-Dix). We compared three specific parameters of four-part Log-Linear equations between the nerve response of control and noise-exposed locusts: Hill coefficient, maximum asymptote, sound pressure level at inflection point; and one general fit, the F statistic. Directly after the first noise exposure, the spiking activity of the auditory nerve is decreased, compared to controls when taking into account the dependence of sound pressure level on the transduction current (Locust condition ∗ SPL interaction) (Figure 4Di, LMEM t_(315)_ = −6.60 p < 0.000001, Cohen’s effect size d (at 90 dB SPL) = −2.04, **F statistic = 392**), but recovers within 24 h (Figure 4Dii, LMEM t_(235)_ = −0.910 p < 0.364, **F statistic = 23**) before once again becoming more distinct 48 h afterward (Figure 4Diii, LMEM t_(316)_ = 5.2, p < 0.00000035, **F statistic = 185**). Directly after the second noise exposure (day 4), the spiking activity of the auditory nerve is decreased (Figure 4Div, LMEM t_(305)_ = −7.28 p < 0.000001, Cohen’s effect size d (at 90 dB SPL) = −1.73. **F statistic = 1161**), and remains lower than controls on day 5 (Figure 4Dv, LMEM t_(295)_ = −3.56 p = 0.000431, **F statistic = 108**) and day 6 (Figure 4Dvi, LMEM t_(235)_ = -3.72 p = 0.000253, Cohen’s effect size d (at 90 dB SPL) = −0.99, **F statistic = 121**). On day 12 after four 12 h noise exposures, the decrease in the sound-evoked nerve response is most severe (Figure 4Dvii, LMEM t_(355)_ = −6.49 p < 0.000001, Cohen’s effect size d (at 90 dB SPL) = −1.73, **F statistic = 269**) before age-related hearing loss starts to narrow the difference between control-aged locusts and noise-exposed-aged locusts (Figure 4viii, ix: Day 18 LMEM t_(235)_ = −3.36 p < 0.0009, Cohen’s effect size d (at 90 dB SPL) = −0.90 **F statistic = 126**; Day 24 LMEM t=_(275)_-3.33 p < 0.00097, Cohen’s effect size d (at 90 dB SPL) = −0.750, **F statistic = 66**).

### Morphology and number of auditory neurons and supporting cells in Müllers organ

**Figure 5:**
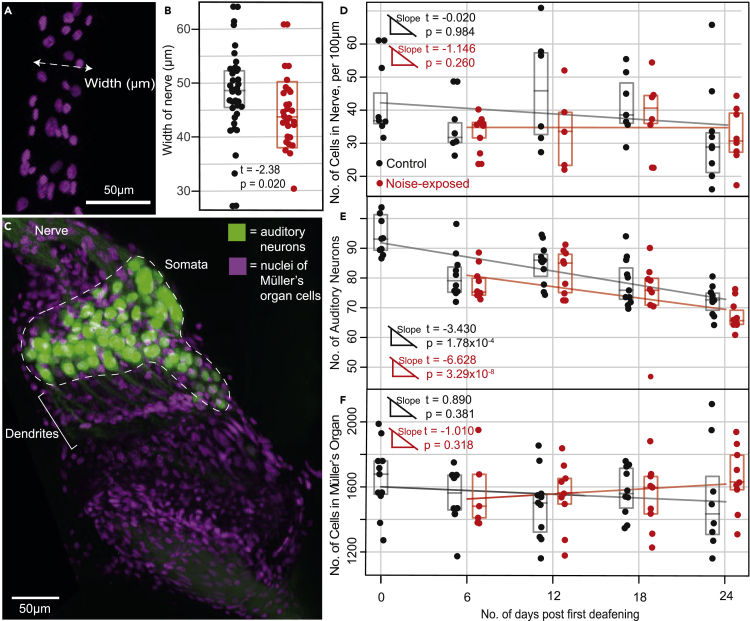
Anatomical analysis of Müller’s organ for noise-exposed and control locusts (A) DAPI (nuceli) staining of cells in auditory nerve showing measurement of nerve width. (B) Comparison of auditory nerve width for noise-exposed and control locusts. Box plots represent Q1, median and Q3. (C) Dual staining of auditory neurons and DAPI staining of the nuclei of all cells in Müller’s organ. (D) Number of cells per 100 μm in the auditory nerve in noise-exposed and control locusts over 24 days. Linear regression analysis output is plotted for each treatment group. (E) Number of auditory neurons in noise-exposed and control locusts’ ears over 24 days. (F) Number of cells in Müller’s organ in noise-exposed and control locusts over 24 days.

We backfilled the auditory nerves of control locusts and noise-exposed locusts *in vivo* with neurobiotin. We then stained auditory nerves with strepavidin florescent dye and the nuclei of all cells with DAPI. We counted the number of auditory neurons and number of cells in Müller’s organ, the density of cells in the auditory nerve, and auditory nerve width. Müller’s organ is composed of six different types of cells: auditory neurons, Schwann cells, fibrous cells, scolopale cells, attachment cells, and hypodermal cells (Figure 2). The auditory nerve width was reduced after noise exposure (Figures 5A and 5B) (Linear Model: t_(61)_ = −2.38, p = 0.0204) but did not decrease as a function of age (Linear model: t_(61)_ = −0.675, p = 0.502). There was no profound decrease in the number of cells in the auditory nerve as a function of age (LM: t_(61)_ = −1.19, p = 0.239) and noise exposure (LM: t_(61)_ = −1.305, p = 0.198). Similarly, the number of cells in Müllers organ did not change as a function of age (LM: t_(77)_ = −0.313, p = 0.755) or noise exposure (LM: t_(77)_ = 0.419, p = 0.676). The number of auditory neurons strongly decreased as a function of age (LM: t_(77)_ = -8.10 p < 0.00001) and a small compounding effect of noise exposure of the number of auditory neurons increased as a function of age (day 6, t_(10)_ = −0.49, day 12, t_(17)_ = −0.91, day 18, t_(16)_ = −1.10, day 24, t_(14)_ = −1.89).

### Electrical properties of auditory neurons and transduction current dependence on sound pressure level

**Figure 6:**
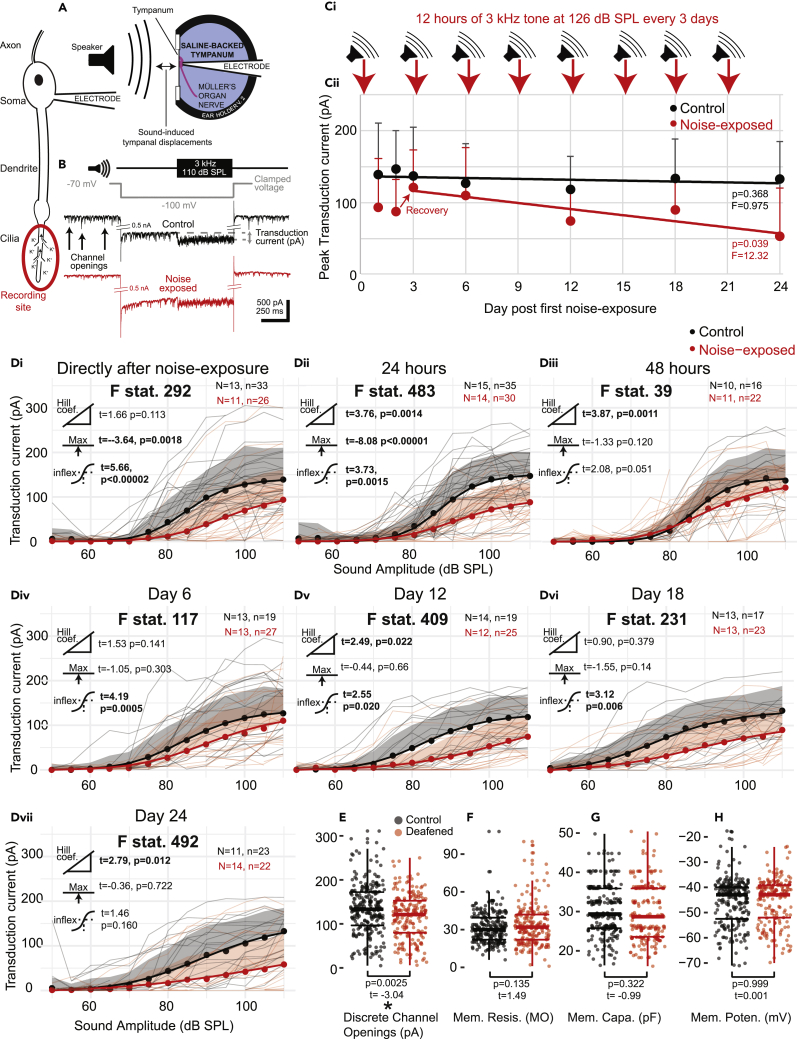
Analysis of tone-evoked transduction currents and electrophysiological properties for individual auditory neurons from noise-exposed and control locust ears (A) Schematic depicting experimental setup. (B) Example of recording of the transduction current, including tone and voltage clamping protocols. (Ci) Experimental workflow of noise-exposure and mock noise exposure (for control locusts). (Cii) Plot of average maximum transduction current for noise-exposed and control locusts for each day. (Di) Plot of the transduction current as a function of SPL of a 3 kHz tone for control and noise-exposed locusts within 12 h after cessation of noise exposure. Transduction currents for individual locusts are plotted as thin gray or thin red lines for control and noise-exposed, respectively. Dots are the average transduction current for each treatment at each SPL and shaded region is positive standard deviation for each treatment and SPL. Thick lines are four-parameter Log-Linear fits for each treatment. The extent of the difference between each group and a statistical test of a difference are displayed as t and p values next to three icons donating (from top to bottom) Hill coefficient, maximum asymptote, and the inflection point. Values are in bold when p < 0.05. The F statistic compares the fit of the model when the treatment of the group (noise-exposed or control) was omitted. Higher F statistics donate a larger effect of treatment. Dii and Diii plot transduction current against SPL for noise-exposed and control locusts 24 and 48 h cessation of noise exposure (or silent speaker for control). Div, Dv, Dvi, and Dvii are 48 h after the 2^nd^, 4^th^, 6^th^, and 8^th^ noise exposure, respectively. (E) Plots the discrete channel opening current amplitude for each auditory neuron for auditory neurons from noise-exposed and control locusts. The extent of the difference and the statistical test for any difference are plotted as t and p values, respectively. (F–H) plots the membrane resistance, membrane capacitance, and membrane potential for each auditory neuron from noise-exposed and control locusts. Linear models were used to test for differences for electrophysiological properties of auditory neurons from control and noise-exposed locusts, with their t and p values displayed on the figures.

We measured the electrical properties and tone-elicited currents of Group III neurons in Müller’s organ through whole-cell patch-clamp recordings from individual neurons from excised ears (Figure 6A). We isolated and optimized the transduction current at the distal ciliated end of the auditory neuron using pharmacology, voltage protocols, and the optimal sound stimulus (Figure 6B) (detailed in methods). We recorded directly after the first 12 h noise exposure at day 1 then 24 and 48 h afterward on day 2 and 3. Recordings at day 6, 12, 18, and 24 were all made 48 h after the last 12 h noise exposure (Figure 6C). The transduction current increased with an increased sound amplitude and followed a log-linear model (Figure 6D). Directly after noise exposure, the maximum transduction current is reduced (Figure 6Di, t=_(17)_-3.64, p = 0.0018, Cohen’s effect size d (at 90 dB SPL) = −1.03) and reduces further 24 h after noise exposure (Figure 6Dii, t=_(17)_-8.08, p < 0.00001 Cohen’s effect size d (at 100 dB SPL) = −1.28). Only 48 h after the first noise exposure is the maximum transduction current comparable with non-noise-exposed controls. Interestingly, the Hill coefficient, which mathematically derives the interaction between sound pressure level and transduction current, does not change directly after noise exposure (Figure 6Di t=_(17)_1.66, p = 0.113), but changed after 24 h (Figure 6Dii t = 3.76, p = 0.0014 and remains different 48 h later (Figure 6Diii, t=_(17)_3.87, p = 0.0011). After two noise exposures and a 48 h recovery (Day 6), both groups are still fairly similar (Figure 6Div). Upon further noise exposure, there is further decline in the maximum transduction current of the noise-exposed group (Figure 6C, Dv-Dvii Cohen’s effect size d (at 110 dB SPL) = −1.24) with the F statistic at its highest (492) when comparing control and noise-exposed locusts at 24 days whereas the control locusts show no age-related decline (Figure 6C, Dv-Dvii).

We recorded smaller discrete channel currents in the auditory neurons of noise-exposed locust auditory neurons compared to control (Figure 6E, LM t_(413)_ = -3.104, p < 0.002, Cohen’s effect size d = −0.306). Discrete depolarizations are assumed to be transient stochastic opening of mechanotransduction ion channels shown both in insects (Hill, 1983; Warren and Matheson, 2018) and vertebrate auditory receptors (Beurg et al., 2015; Pan et al., 2013). There were no differences in the membrane resistance, membrane capacitance, and membrane potential (at zero holding current) for the auditory neurons of control and noise-exposed locusts (Figures 6F, 6G, and 6H). However, there was a trend for the resting potential of auditory neurons of aged and deafened locusts to be over 7 mV depolarized over 24 days of aging (LM; t = 3.345, p = 0.001).

## Discussion

Repeated or prolonged noise exposure causes hearing loss which, for humans, has the largest difference from non-noise-exposed individuals during the middle of their life (Passchier-Vermeer, 1974). As humans age, age-related hearing loss dominates and noise exposure plays less of a role in determining hearing loss (Corso, 1980; Echt et al., 1998). The interaction of age and noise on hearing loss is well known for the human auditory system thanks to extensive longitudinal audiometric measurements including from industrial workers before ear protection (Passchier-vermeer, 1968; Corso, 1963). Here, we repeatedly exposed large numbers of locusts to noise and quantified their ability to hear over their life by measuring the response of their auditory nerve to sound. We also measured components of the auditory system known to correlate with hearing loss: decrease in auditory receptors, auditory nerve thinning, decrease of electrochemical gradients, and other components crucial to the function of the auditory system: supporting cells, auditory receptor electrophysiological properties, and transduction. We found striking parallels with the pattern of human hearing loss and rigorously quantify how noise and age affect specific components in the ear.

Before sound can impinge on delicate sensory structures within an auditory system, it is captured mechanically through antennae or tympani. In response to noise, the tympanal displacements of the desert locust transiently decrease 24 h after noise exposure but remain unaffected directly after or 48 h after noise exposure. In the fruit fly, noise up-shifts and sharpens the mechanical tuning of its sound-capturing antennae (Boyd-Gibbins et al., 2021). This is due to changes in the active mechanical output of its motile auditory receptors. Although the locusts’ tympanum does not actively vibrate, there is evidence that the state of auditory neurons can influence its mechanical properties (Möckel et al., 2007). There are clear increases in the expression of endocuticle structural proteins in the locust ear, directly after a 24 h noise exposure (twice as long as used here) (French and Warren, 2021) which could presumably influence tympanal displacments. Temporary mechanical remodeling of the tympanum is unlikely to explain the transient decrease in tympanal displacements and we suggest that changes in Müller’s organ itself, perhaps even in the auditory receptors, would best explain this finding. Mammals, by comparison have incredibly robust mechanical elements (ossicles) to transfer sound to their auditory systems (Thompson et al., 1979; Wada et al., 1994; Holte, 1996). It appears that the mechanical operation of the locust’s tympanum is also fairly robust as control and noise-exposed tympani are indistinguishable directly after and 48 h later but further longitudinal measurements are required to confirm this.

We measured compound spiking potentials from the auditory nerve in response to a 3 kHz pure tone at SPL (Sound Pressure Levels) from 20 to 110 dB. We found a decrease in maximal nerve response directly after noise exposure (Figure 4Di, Div). This is similar to a decrease in nerve response in *Drosophila* (Boyd-Gibbins et al., 2021) and the first wave of auditory brainstem responses (ABRs) and compound action potentials after noise exposure in mice (Kujawa and Liberman, 2009; Chuang et al., 2014) and rats (Tagoe et al., 2014). Noise-exposed locusts recovered their maximal nerve response within 24 h after first noise exposure but then the auditory nerve response decreased again at 48 h later (Figure 4Di-Diii), a 48 h pattern repeated after the second noise exposure on day 4 (Div-Dvi). By comparison, mice (with an accelerated hearing loss phenotype) recover quickest in the first three days before their recovery slows. There is no sign of a reversal of recovery of hearing function in mice but this is limited to the time points measured after noise exposure: straight after, 1, 3, 7, and 14 days (Chuang et al., 2014) and 24 h, 8 days, and 15 days (Milon et al., 2021). Other studies that measured hearing loss after noise exposure have only ever found recovery (Li and Borg, 1993; Howgate and Plack, 2011; Chuang et al., 2014; Fernandez et al., 2015; Kujawa and Liberman, 2009). Although the reversal of recovery found for locusts appears paradoxical, no study has measured recovery twice within 48 h to uncover short-term changes. This pattern of recovery mirrors some traumatic brain injuries where initial recovery can be followed by worsening of symptoms. The physiological basis of such non-linear recovery is due to different etiologies presenting over distinct time courses. In the case of Traumatic Brain Injury (TBI), initial electrophysiological dysfunction is followed by structural damage then toxic-metabolic and vascular effects (Giza and Hovda, 2001). We suggest that noise exposure has a complex recovery process where an initial promising recovery is followed shortly after by deterioration and is the result of multiple processes, triggered by noise exposure, acting over distinct time courses. Supporting this interpretation, there are changes in gene expression triggered after noise exposure in mice that show delayed upregulation. Mice exposed to noise show transient upregulation at 24 h (compared to 6 h and 2 days after) of the *Aft* gene family (iron activated transcription factor) in spiral ganglion neurons and the transcriptional changes after noise display different unique patterns (Milon et al., 2021).

The long-term effects of repeated noise exposure result in a decreased maximum nerve response and higher SPL at the inflection point (indicative of increased hearing thresholds). But age-related hearing loss leads to narrowing of the differences between control and noise-exposed locusts at older ages. This follows human hearing loss the only other organism with a high enough powered comparative longitudinal dataset (Corso, 1980) and appears to be the same for mice (Alvarado et al., 2019) and suggests this pattern of degeneration is fundamental across diverse biological auditory systems. If this proves to be true, we need to understand processes shared across anatomically different auditory systems to uncover their physiological basis.

Auditory receptor number decreased steadily due to age with a mild compounding effect of noise. In fruit flies, not exposed to noise, auditory receptor number remains stable across most of its life course (Keder et al., 2020). It is surprising to find no age-related changes in auditory neuron number in flies but a decrease in locusts—two distinct animals, and auditory systems, within the same taxonomic class (Insecta). In mammals, auditory receptor number is reduced due to age in the apical (low frequency) part of the cochlea and due to noise in the basal (high frequency) part of the cochlea (Wu et al., 2020; Dixon and Arndt, 1971; Clark et al., 1974; Moody et al., 1978; Bohne and Harding, 2000; Chen and Fletcher, 2003). The majority of auditory neurons counted here are sensitive to mid-frequencies but an (ongoing future) analysis of the low- and high-frequency Group I and II auditory neurons will determine if this frequency-age-noise-specific pattern of auditory receptor loss is a general feature of auditory systems. We expect that higher frequency auditory receptors, across animals, are more vulnerable to age and noise deficits due to higher metabolic demand to maintain their higher endolymph potential.

The myelin sheath encapsulating auditory nerves of mammals is damaged after noise exposure (Pilati et al., 2012; Tagoe et al., 2014; Wan and Corfas, 2017) and auditory nerve thinning in locusts with noise suggests that sound propagation along axons represents a similar vulnerability for the locust auditory system. The number of non-sensory cell types has not been counted in any auditory system and we found no decrease in non-neuronal cell types, either in the nerve or Müller’s organ, in the locusts either as a function of noise or age.

In individual auditory neurons of Müller’s organ, membrane resistance and cell capacitance were unaffected either by noise or age. There was a trend for older auditory neurons (irrespective of treatment) to be more depolarized ∼3 mV, although the magnitude of the change is small (t = −2.382, p = 0.0177). While there are no changes in the membrane resistance and membrane potential of hair cells of mice, their capacitance decreased as a function of age (Jeng et al., 2020). Properties of the transduction current in mice were unaffected by age which matches that found here for auditory neurons of locusts. This reveals remarkable homeostatic mechanisms at play to maintain auditory receptor function. Repeated exposure to noise, however, results in a steady decrease in the electrochemical driving force for the transduction current, hypothesized to be responsible for a decrease in the transduction current (Warren et al., 2020). In mammals, the electrochemical driving force for the scala media is established by specialized ion-pumping cells in the stria vascularis. Dysfunction of the stria vascularis has been recently popularized as responsible for the majority of age-related hearing loss (Ramadan and Schuknecht, 1989; Vaden et al., 2017) supported by a downregulation of potassium transport genes in the lateral wall (Milon et al., 2021). However, physiological analysis (Wu et al., 2020) suggests that although the correlation of stria vascularis atrophy in patients with hearing loss is high (Schuknecht, 1993; Schuknecht et al., 1974), it does little to predict the extent of hearing loss.

Some components in the locust ear are vulnerable to noise (electrochemical gradient, auditory nerve), others age (auditory neuron number), yet other components are incredibly robust to age and noise (supporting cells). On top of this layer complexity are compounding interactions where degeneration due to noise is compounded by age (auditory nerve thickness) or where the damage caused by noise only becomes apparent after aging (auditory neuron resting potential). We have captured and quantified this complexity in a single auditory system. We therefore predict that the middle-life pattern of hearing loss experienced by noise-exposed humans is due to a complex interaction of multiple components.

Despite the clear anatomical differences in auditory systems of insects and vertebrates, they share equivalent components: transduction on membrane protrusions (microtubule-based cilia in insects and actin-based villi in mammals), electrochemical gradients across the auditory receptors, and spiking axons/dendrites that carry auditory information to the central nervous system. In addition, they share developmentally interchangeable transcription factors for ear development (Wang et al., 2002) and evolved from the same ancestral ciliated sensory appendage, sharing ∼1,500 million years of evolution before they split apart ∼600 million Years Ago (Nowotny and Warren, 2021). As such, insects are a valuable resource to derive general principles of how auditory systems are affected by age and noise. This is keenly evidenced by similarities in the pattern, causes, and consequences of hearing loss across animal phyla despite their stark anatomical differences.

### Limitations of the study

Although we attributed deficits due to age and noise, or a combination of both, we were not able to disentangle a compound effect of age on a noise deficit such as auditory nerve thinning. For instance, the auditory nerve was thinner 72 h after the first noise exposure and this difference increased for the next 21 days. But the noise-exposed locusts continued to be exposed to noise every three days as they aged. To confirm a true compounding effect of age, on a deficit due to noise, locusts would need to be exposed once only and then aged. Not all cell types and processes affected by age and noise are comparable between diverse auditory organs. For instance, the loss of auditory receptors in mammals happens due to age but also noise, whereas for the desert locust it is purely dependent on age. We focused on one group of auditory neurons which are frequency tuned to 3 kHz as this is what the majority of auditory neurons in Müller’s organ are tuned to. Our analyses did not analyze the frequency-specific nature of hearing loss.

## STAR★Methods

### Key resources table

**Table:** 

REAGENT or RESOURCE	SOURCE	IDENTIFIER
**Deposited data**		

Analysed data sets	Mendeley	https://data.mendeley.com/datasets/yc8m5wpykt/2
RStudio code for figure preparation and statistics	Mendeley	https://data.mendeley.com/datasets/yc8m5wpykt/2

**Experimental models: Organisms/strains**		

*Schistocerca gregaria* (phase gregaria)	University of Leicester Locust Labs	Please contact Ben Warren, bw120@le.ac.uk

**Software and algorithms**		

RStudio Version 1.4.1106	RStudio Team (2021)	https://www.rstudio.com
Rstudio package *LME4*	Bates et al., 2015	https://cran.r-project.org/web/packages/lme4/index.html
RStudio package *simr*	Green and Macleod, 2016	https://cran.r-project.org/web/packages/simr/index.html
Rstudio package lmerTest	Kuznetsova et al., 2017	https://cran.r-project.org/web/packages/lmerTest/index.html
Rstudio package *drc*	Ritz and Strebig, 2016	https://cran.r-project.org/web/packages/drc/index.html
Rstudio package *anova*	Ritz et al., 2015	https://cran.r-project.org/web/packages/easyanova/index.html
Igor Pro 6.3.7.2	WaveMetrics Inc.	https://www.wavemetrics.com
Matlab R2018a	Mathworks	https://www.mathworks.com
ImageJ 1.53c	Wayne Rasband, National Institutes of Health	http://imagej.nih.gov/ij
Imaris 9.9	Oxford Instruments Group	https://imaris.oxinst.com

### Resource availability

#### Lead contact

Further information and requests for resources and data should be directed to and will be fulfilled by the lead contact, Ben Warren (bw120@le.ac.uk).

#### Materials availability

• *Schistocerca gregaria* used in these experimental are from the Leicester Locusts labs colony (populated from eggs collected from Mauritania in May 2015). We are open to share our locust strain with other researchers.
• This study did not generate new unique reagents.

### Experimental model and subject details

#### The desert locust

We raised *Schistocerca gregaria* (Mixed sex) in crowded conditions 150–250 in 60 cm^3^ cages in their fast-aging gregarious state, where they can live up to two months. This contrasts with their isolated solitarious state where they live for up to nine months. *S. gregaria* where fed *ab libitum* fresh wheat (grown in house) and milled bran. The founding progeny of the Leicester Labs strain were solitary copulating adults collected at Akjoujt station ∼250 km North East from Nouakchott, Mauritania in May 2015.

### Method details

#### Noise exposure and acoustic stimulation

No anaesthesia was used for experiments with locusts. The wings of all locusts (control and noise-exposed) were cut off at their base to increase noise exposure of the conditioning tone to their tympanal ears, which are otherwise covered by their wings. Between ten and twenty locusts, for both the noise-exposed group and the control group, were placed in a cylindrical wire mesh cage (8 cm diameter, 11 cm height). Both cages were placed directly under a speaker (Visaton FR 10 HM 4 OHM, RS Components Ltd). For the noise-exposed group only, the speaker was driven by a function generator (Thurlby Thandar Instruments TG550, RS Components Ltd) and a sound amplifier (Monacor PA-702, Insight Direct Ltd) to produce a 3 kHz tone at 126 dB SPL (Sound Pressure Level), measured at the top of the cage where locusts tended to accumulate. Throughout the paper we refer to noise that the locusts are exposed to as a 3 kHz 126 dB SPL pure tone. This tone was played continuously for 12 h overnight (21:00-09:00) for the noise-exposed group during their natural darkness period. The control group was housed in an identical cage with a silent speaker for 12 h. All recordings were performed within a 12 h window during the day. Work conducted by Megan Barnes found that a 126 dB SPL 3 kHz tone at 30 min, 3 and 6 h did not cause a stark decrease in the sound-evoked nerve response. Sound Pressure Levels (SPLs) were measured with a microphone (Pre-03 Audiomatica, DBS Audio) and amplifier (Clio Pre 01 preamp, DBS Audio). The microphone was calibrated with a B&K Sound Level Calibrator (CAL73, Mouser Electronics). For laser measurements, hook electrode and patch-clamp recordings, the locust ear was stimulated with the same speaker and amplifier as above with a 3 kHz pure tone duration of 0.5 s. For hook electrode recordings the 3 kHz tone had a rise and fall time of 2 ms. Tones were played three times for each locust at each SPL and the average response taken for each SPL. For intracellular recordings from individual auditory neurons the speaker was driven by a custom made amplifier controlled by an EPC10-USB patch-clamp amplifier (HEKA-Elektronik) controlled by the program Patchmaster (version 2 × 90.2, HEKA-Elektronik) running under Microsoft Windows (version 10).

#### Biomechanical measurements of the tympanum with laser Doppler vibrometry

For *in vivo* measurements of the tympanum, locusts were mounted in natural dorso-ventral orientation following removal of wings and hind legs, and fixed to a copper platform using blue tac wrapped around their thorax and abdomen (Bostik, Colombes, France). Once secured, with their metathoarcic legs removed the locusts tended not to struggle further. They were positioned so that their tympanum was perpendicular to the micro-scanning Laser Doppler Vibrometer (PSV 500 with close up unit and 150 mm lens, Polytec, Waldbronn, Germany). We used the inbuilt low-pass filter of the Polytec software to filter out slower movements of the tympanum due to breathing-related movements. The stimulus was produced with a waveform generator (SDG 1020, Siglent, China), and delivered via a stereo amplifier (SA1 power amplifier, Tucker-Davis Technologies, Alacchua, Florida) to a loudspeaker (MF1, Tucker-Davis Technologies, Alacchua, Florida) positioned 15 cm away from the animal to avoid nearfield measurement. The stimulus amplitude was obtained by manually changing the voltage within the waveform generator, and recording the SPL using a 1/8ʺ microphone (Type 4138, Brϋel & Kjaer, Germany) with built in preampflifer (B&K 2670, Brüel & Kjær, Denmark), calibrated using a sound-level calibrator (Type 4237, Brϋel & Kjaer, Denmark), via a conditioning amplifier (Nexus 2690-OS1, Brüel & Kjær, Denmark). Displacement data of tympanum vibration was collected using the PSV internal data acquisition board with a 128 ms sample length at a sampling rate of 512 kHz, averaged 10 times per sample. Locust positioning and measurements were carried out within <5 min for each locust. Experiments were carried out in an acoustic booth (IAC Acoustics, Series 120a, internal dimensions of 2.8 × 2.7 × 2 m) on a pneumatic vibration isolation table (Nexus Breadboard (B120150B), 1. 2 × 1.5 × 0.11 m, Thor Labs, USA). Displacements were measured as the average peak-to-peak displacement.

#### *In vivo* hook electrode recordings from auditory nerve six

Locusts were secured ventral side up with their thorax wedged in a plasticine channel and their legs splayed and held down with plasticine. A section of the second and third ventral thoracic segment was cut with a fine razor blade and removed with fine forceps. Tracheal air sacks were removed to expose nerve six and the metathoracic ganglia. This preparation left the abdomen, including the 1^st^ segment where the ears reside intact. Thus, maintaining the operation of the ear *in vivo*. Hook electrodes constructed from silver wire 18 μm diameter (AG549311, Advent Research Materials Ltd) were hooked under the nerve and the nerve was lifted out of the haemolymph. A mixture of 70% Vaseline and 30% paraffin oil was applied through a syringe to coat the auditory nerve to stop it drying out. Locust mounting and recordings took ∼15 min for each locust. Signals were amplified 1,000 times by a differential amplifier (Neurolog System) then filtered with a 500 Hz high pass filter and a 50 kHz low pass filter. This amplified and filtered data was sampled at 25 kHz by Spike2 (version 8) software running on Windows (version 10). To quantify the compound spiking activity of the auditory nerve we used Matlab (Version R2020a, Mathworks Inc.) and rectified the nerve signal and integrated the area underneath. We computed this for the 0.5 s of sound-evoked neural activity and for 60 s background nerve activity before the tones and the background activity between the tones. To compute the σ ratio we divided the sound-evoked response by the background neural activity. N.B. the locust treatment was blinded to the experimenter until all data was collected and analysed.

#### Dissection of Müller’s organ and isolation of Group-III auditory neurons

Whole cell patch clamp recordings per performed on group-III auditory neurons because they form the majority of auditory neurons of Müller’s organ (∼46 out of ∼80) (Figure 2A) (Jacobs et al., 1999), they are the most sensitive auditory neurons of Müller’s organ (Römer, 1976) and are broadly tuned to the 3 kHz we used for noise-exposure (Warren and Matheson, 2018). For intracellular patch-clamp recordings from individual auditory neurons the abdominal ear, including Müller’s Organ attached to the internal side of the tympanum, was excised from the first abdominal segment, by cutting around the small rim of cuticle surrounding the tympanum with a fine razor blade. Trachea and the auditory nerve (Nerve 6) were cut with fine scissors (5200-00, Fine Science Tools), and the trachea and connective tissue removed with fine forceps. This preparation allowed perfusion of saline to the internal side of the tympanum, necessary for water-immersion optics for visualizing Müller’s Organ and the auditory neurons to be patch-clamped, and concurrent acoustic stimulation to the dry external side of the tympanum. The inside of the tympanum including Müller’s Organ was constantly perfused in extracellular saline. Dissection, protease and recordings took ∼60 min for each locust ear.

To expose Group-III auditory neurons for patch-clamp recordings, a solution of collagenase (0.5 mg/mL) and hyaluronidase (0.5 mg/mL) (C5138, H2126, Sigma Aldrich) in extracellular saline was applied onto the medial-dorsal border of Müller’s Organ through a wide (12 μm) patch pipette to digest the capsule enclosing Müller’s Organ and the Schwann cells surrounding the auditory neurons. Gentle suction was used through the same pipette to remove the softened material and expose the membrane of Group-III auditory neurons. The somata were visualized with a Cerna mini microscope (SFM2, Thor Labs), equipped with infrared LED light source and a water immersion objective (NIR Apo, 40×, 0.8 numerical aperture, 3.5 mm working distance, Nikon) and multiple other custom modifications. For a full breakdown of the microscope components and how to construct a custom patch-clamp microscope for ∼£12k see: https://www2.le.ac.uk/departments/npb/people/bw120.

#### Electrophysiological recordings and isolation of the transduction current

Electrodes with tip resistances between 3 and 4 MΩ were fashioned from borosilicate class (0.86 mm inner diameter, 1.5 mm outer diameter; GB150-8P, Science Products GmbH) with a vertical pipette puller (PC-100, Narishige). Recording pipettes were filled with intracellular saline containing the following (in mM): 170 K-aspartate, 4 NaCl, 2 MgCl2, 1 CaCl2, 10 HEPES, 10 EGTA. 20 TEACl. Intracellular tetraethylammonium chloride (TEA) was used to block K^+^ channels necessary for isolation the transduction. To further isolate and increase the transduction current we also blocked voltage-gated sodium channels with 90 nM Tetrodotoxin (TTX) in the extracellular saline. The addition of ATP to the intracellular saline did not alter the electrophysiology of the recordings so was omitted. During experiments, Müller’s Organs were perfused constantly with extracellular saline containing the following in mM: 185 NaCl, 10 KCl, 2 MgCl2, 2 CaCl2, 10 HEPES, 10 Trehalose, 10 Glucose. The saline was adjusted to pH 7.2 using NaOH. The osmolality of the intracellular and extracellular salines’ were 417 and 432 mOsm, respectively.

Whole-cell voltage-clamp recordings were performed with an EPC10-USB patch-clamp amplifier (HEKA-Elektronik) controlled by the program Patchmaster (version 2 × 90.2, HEKA-Elektronik) running under Microsoft Windows (version 7). Electrophysiological data were sampled at 50 kHz. Voltage-clamp recordings were low-pass filtered at 2.9 kHz with a four-pole Bessel filter. Compensation of the offset potential were performed using the “automatic mode” of the EPC10 amplifier and the capacitive current was compensated manually. The calculated liquid junction potential between the intracellular and extracellular solutions was also compensated (15.6 mV; calculated with Patcher’s-PowerTools plug-in from www3.mpibpc.mpg.de/groups/neher/index.php?page=software). Series resistance was compensated at 77% with a time constant of 100 μs.

#### Staining and confocal microscopy

Locusts were secured ventral side up in plasticine. A square section of the second and third ventral thoracic segment was cut with a fine razor blade and removed with fine forceps and set aside. Tracheal air sacks were removed to expose nerve six and the metathoracic ganglia. The thoracic cavity was filled with locust extracellular saline and auditory nerve six was pinched and broken at the top of the nerve, close to the metathoracic ganglion with fine forceps and the cut end of nerve six placed on the thoracic cuticle outside the thoracic cavity at the posterior end. A well was formed around the nerve end with petroleum jelly (Vaseline, Boots) using a syringe (1.1 mm diameter). The well was filled with Neurobiotin (5% m/v, SP-1120, Vector Laboratories) dissolved in distilled water before a lid was fashioned with more petroleum to seal the well. The square of thoracic cuticle was replaced onto the locust thoracic cavity and sealed back in place with petroleum jelly, to prevent desiccation. Locusts were incubated overnight at 4°C to allow Neurobiotin to diffuse along the nerve and fill the auditory neurons of Müller’s organ. Following overnight incubation, whole ears were excised from the first abdominal segment, as described above. Whole locust ears were fixed in 4% paraformaldehyde (P6148, Sigma Aldrich) dissolved in Phosphate Buffer Saline (PBS) for 24 h at 4°C, in a single well of a 24 plastic-well plate. Following fixation, ears were washed in PBS (3 × 10 min) at room temperature, before being stored in PBS at 4°C. Locust ears were washed in PBS, with 0.2% m/v Triton X-100 and 5% Normal Goat Serum (m/v) (S26-LITER, Merck Life Science UK LTD) (PBST.NGS) for 3 × 2 h on an orbital shaker (160 rpm) at room temperature. Ears were then gently shaken (120 rpm) at 4°C overnight in 20 μg/mL Dylight 488 strepavidin (SA-5488, Vector Laboratories) and 0.05 mg/mL DAPI (D9542, Sigma Aldrich), diluted in PBST.NGS. During this time the fluorescent strepavidin binds very tightly to the fixed neurobiotin to specifically stain the recorded neurons and DAPI specifically binds to cell nuclei. After this overnight incubation, Müller’s organs were washed three times in PBS (3 × 10 min), dehydrated in an ethanol series and cleared in Methyl salicylate (M6752, Sigma Aldrich).

Fluorescence images (pixel size 0.31 μm^2^, with a total of 65 z stacks) were captured with a confocal microscope (FV1000 CLSM, Olympus) equipped with Plan-UPlanSApo 20× (0.75 numerical aperture) lens. Fluorescence emission of Dylight 488 was collected through a 505–530 nm bandpass filter and fluorescence emission of DAPI was collected through a 485 nm low pass filter. Confocal images were adjusted for contrast and brightness, overlaid and stacked in ImageJ (version 1.51, National Institutes of Health).

#### Morphological analysis and cell counting of Müller’s organ

To count the auditory neurons, a maximum intensity z-projection was produced for each z-stack image in ImageJ, displaying the auditory neurons as a 2D image. Neurons were manually counted using the Cell Counter plugin (Kurt de Vos, University of Sheffield, https://imagej.nih.gov/ij/plugins/cell-counter.html). Contrast/brightness parameters were adjusted to ensure all neurons were counted.

Imaris software was used for automated cell counts of the DAPI stained nuclei present in Müller’s organ and nerve. Automated cell counting was performed on 3D images using the “Spots” tool, with consistent parameters (15 μm spot size) used across all Müller’s organs. The “Surfaces” tool was used to ensure only cells within the Müller’s organ were included in the automated counting. The width of the auditory nerve was also measured in Imaris using the MeasurementPro tool.

### Quantification and statistical analysis

We designed all experiments to have a power above 95%, which gives an ability to detect a difference between control and noise-exposed locusts of 95% (if these series of experiments were run an infinite amount of times). Our false negative rate, or type II error probability is <5% (1-power) (probability of not finding a difference that is there). Our false positive rate or type II error (probability of finding a difference that is not there) is determined by our p values which was set at 0.05. In order to calculate the power we used the raw data, and the effect size, reported in Warren et al. (2020) for: Tympanal displacements measured with Doppler laser vibrometry (Figure 1Ai), hook electrode recordings of tone-evoked potentials from the auditory nerve (Figure 2) and whole-cell patch-clamp recordings from individual auditory neurons of Müller’s organ (Figure 3I). There exists no analytical methodology for conducting power calculations on Linear Mixed Effect Models (LMEM). Therefore we generated a dataset simulated from the raw data of Warren et al. (2020), fitted a Linear Mixed Effect Model, then ran repeated simulations of the LMEM 1000 times. We used the proportion of time that the LMEM reported a difference to calculate the power. For this paper the experiments that measured differences in tympanal displacements, hook electrode responses and the transduction current all had at least 95% power when using n numbers of 16, 9 and 12 respectively. This simulated power analysis also, only applies for the effect size reported in Warren et al. (2020) and may be lower if the actual effect size, in this paper, is reduced. This is especially true for tympanal displacement measurements which were only ∼ twice as different in these recordings as opposed to ∼10 times different in Warren et al. (2020). Models were fitted in R (Version 2.4.3), on a Windows PC running Windows 10 using the package *LME4* (Bates et al., 2015) and simulations were run with the package *simr* (Green and Macleod, 2016).

To calculate the number of locusts required to maintain a power of 95% for Müller’s organ morphological analysis we used preliminary data collected by Georgina Fenton. Georgina found an effect size *d* of 1.45 between the number of cells in Müller’s organ at 10 days old and 38 days old (two independent groups). We used G∗Power t-test *A Priori* power analysis to calculate that a total sample size of 24. We settled on a sample size of 20, which still achieves a power of at least 95% when regression analysis across all five time points are taken into account (e.g. at days: 0, 6, 12, 18, 24 Figure 1B).

Throughout the manuscript n refers to the number of recorded neurons and N refers to the number of Müller’s Organ preparations used to achieve these recordings (i.e. n = 10, N = 6 means that 10 neurons were recorded from 6 Müller’s Organs). All n numbers are displayed on the figures for clarity. The Spread of the data is indicated by 1 standard deviation as the standard deviation indicates the spread of the data, unlike standard error. Median and Q1 and Q3 are displayed by bars when individual measurements are plotted. For all hook electrode recordings, 80% of patch-clamp recordings and 50% of neurobiotin backfills the treatment of the locust (noise-exposed or control) was blinded to the experimenter; lone working conditions, due to Covid restrictions, made complete blinding impossible. All data either remained blinded or was recoded to be completely blind when analysing the data to avoid unconscious bias.

To test for differences and interactions between control, noise-exposed and aged locusts we used either a linear model (LM) or Linear Mixed Effects Model (LMEM), with treatment and age as fixed effects, and Locust identity and SPL as a random intercept, when repeated measurements are reported. Models were fitted in R (Version 3.4.3) with the package *LME4* (Bates et al., 2015). The test statistic for these analyses (t) are reported with the degrees of freedom (in subscript) and p value, which are approximated using Satterthwaite equation (lmerTest package) (Kuznetsova et al., 2017). We report Cohen’s d effect size for significant differences. Curves where fitted to the data using Matlab (version R2018a) for hook electrode recordings or the *drm* package in R for patch-clamp recordings (Ritz, 2016). The *drm* package was also used to compute t and p values when comparing control and noise-exposed four part Log-Linear models. F statistics of the Log-Linear model fits were computed by excluding treatment (noise-exposed or control) as a factor. Higher F statistics donate a stronger effect of treatment.

In order to compare responses between noise-exposed and control locusts across SPLs we adopted an approach first implemented in pharmacology research. In our work the “dose-response curves” are equivalent to SPL-auditory response curves. This allowed us to maximise the information contained in each dataset and to quantitatively compare model parameters such as: Hill coefficient (steepness of slope), maximal asymptote (maximum σ ratio), and inflexion point (σ ratio at the steepest part of the slope). We did this using the *drm* function of *drc* package (Version 3.1-1, Ritz et al., 2015).

We fitted four-part Log-Linear models with auditory nerve responses (σ ratio) as the dependent variable with treatment (control or noise-exposed) and SPL as the independent variables. This analysis was done for each day and the t and p value reported for each model parameter: Hill coefficient, maximal asymptote and inflexion point on each graph in Figures 2 and 3. The equation of the four parameter log linear fits is:

$$\;Y=c+\frac{d-c}{1+\exp\left(b\left(X-e\right)\right)}$$

Where *Y* is the σ ratio, *b* is the slope at the inflexion point, *c* is the lower asymptote, *d* is the higher asymptote, *e* is the SPL (or *X* value) producing a response halfway between *b* and *c*.

To test whether the factor of treatment (noise-exposed or control) significantly affected auditory nerve response we compared the above model to a model in which treatment was omitted as an independent variable, using the *anova* function (Ritz et al., 2015). This gave an F statistic labelled on each graph in Figures 4 and 6. The p value for this analysis remained below 0.001 for all graphs. We used identical analysis for whole-cell patch-clamp data where transduction current was the dependant variable.

Please let Ben Warren know if you require the raw data and especially if you wish to do a quantitative analysis of sex differences in hearing loss.

## Data Availability

• All analysed data sets have been deposited at Mendeley and are publicly available as of the date of publication (https://data.mendeley.com/datasets/yc8m5wpykt/2). Accession numbers are listed in the key resources table. The DOI is listed in the key resources table. Raw data reported in this paper will be shared by the lead contact upon request within two working weeks as the file sizes are too large to deposit online. • All original code has been deposited at Mendeley and is publicly available as of the date of publication (https://data.mendeley.com/datasets/yc8m5wpykt/2). DOIs are listed in the key resources table. • Any additional information required to reanalyze the data reported in this paper is available from the lead contact upon request.
